# 

**Published:** 1851-10-01

**Authors:** 


### To Correspondents.

ALTHOUGH we have given in this number two sheets and a half of additional matter, we have been reluctantly compelled to lay aside many valuable communications intended for immediate publication. They will appear in January, with a full analysis of Dr. Carpenter's Physiology and the French and German Psychological Journal, &c.

No. II. of the Journal will be reprinted.

### FRENCH LUNATIC ASYLUMS.

Our able, intelligent, and indefatigable correspondent, Dr. Webster, has recently returned from a tour in the north and north-east of France. Having personally inspected the principal provincial lunatic asylums in that part of the continent, we feel much pleasure in announcing that the account of his visit will certainly appear in the next number of our Journal.

We are much indebted to Dr. Parrish, of Philadelphia, for several copies of the "Pennsylvania Journal of Prison Discipline," &c., which will be duly noticed, with the "American Journal of Insanity," and other works forwarded to the publisher.

As it is the wish of the editor of this Journal to make it a medium for the dissemination of *psychological intelligence*, he will be much obliged to his readers for any communications they may do him the favour of forwarding, having reference to the erection of new lunatic asylums, the appointment of medical superintendents, and other facts likely to prove interesting to those engaged in the treatment of the insane.

* Insanity?—*Printer's Devil.*

### INDEX TO VOL. IV.

ABSTINENCE, extreme, effects of, 98, 99.
Acherly, Lieut. C. H., case of, 274.
Admissibility of evidence of lunatics, 436.
Affusion, cold, used as a punishment in French asylums, 145.
Amusements, popular, indicate the intellectual state of a people, 11.
Anæsthesia, moral, illustration of, 593.
Animals have no articulate speech, 406.
——— instinctive ingenuity of, not progressive, 309.
Anatomy, morbid, of the brain, by Holmes Coote, 236.
Apoplexy, sanguineous, rare in Bethlehem, 237.
Appeal from Bethlehem, 231.
Appearances, morbid, commonly observable within the crania of lunatics, 392.
Appetites, morbid, of females, 30.
——— of animals changed by domestication, 92, 93.
Appearance, manly, attractive to the female, 24.
Asylums, pauper lunatic, in Ireland, 83, 521.
——— private lunatic, in Ireland, 89.
——— in France, (from Notes of Dr. Webster,) 127.
——— in America, (from Notes by J. Ball, Esq.,) 429.
——— at Dundrum, for criminal lunatics, 526.
——— lunatic, mortality in, 259, 457.
——— in Ireland, fifth general report of, 521.
——— British, analysis of reports of, 536—574.
——— public, for the insane of the middle classes, Dr. H. Monro on, 594.
——— central, for criminal lunatics, 625.
Association of medical officers of hospitals for the insane, 620.
——— adjourned meeting of, at Colney Hatch, and other institutions, 624, 625.
Atkinson, Henry G., on the nature and laws of man's development, 303.
Aura cerebral, mesmeric theory of, 312.
Aztec children, habits and mental character of, 376.
Ballads and songs, influence of, 74.
Bethlem Hospital, exemption of, from cholera in 1849, 142.
——— history and details of, 275.
——— post-mortem examinations at, 386.
Bennett's, Dr., clinical lectures on nervous diseases, abstract of, 266.
Births, illegitimate proportion of, in different countries, 46.
Blood, abnormal conditions of, impair the sensorial functions, 384.
Brain, proportion of, in man and in the inferior animals, 319.
——— inflammation of, in relation to insanity, 348.
——— morbid anatomy of, by Holmes Coote, 383.
——— slight injuries of, sometimes repaired, 384.
——— certain injuries of, produce weakness of mind not amounting to insanity, 384.
——— ramollissement of, 458.
——— case of hardening of, 240.
——— morbid condition of, causes insensibility to diseases of the body, 385.
Breeding, cross, beneficial effects of, 57.
Browne, Dr., on moral insanity, 555.
Cabanis, opinion of, on the acute perceptions of woman, 22.
Calmness, abnormal, a dangerous symptom, 410, 411.
Cannabis Indica (or hachish), effects of on the mind, 110.
Carpenter, Dr., on alcoholic liquors, 89.
Case of Mr. Dyce Sombre, 154; of a female convulsionist, 209; of Mr. Pearce, 232; of post-mortem examination of the insane, 236, 385; of Lieutenant Acherly, 274; of manslaughter in an asylum, 279; of disputed will (Dryden *v.* Fryer), 285; of admirable presence of mind, 414; of self-mutilation, 452; of ramollissement of the brain, 458; of homicidal impulse, 560; of M'Naughten, 578; of Oxford, 580; of John Lynch, 583; of Anne Valby, 593.
Cases of prolonged abstinence, 98.
Causes of suicide in France, 246, 421.
Chatelet (Du), on the causes and prevention of prostitution, 44, 45.
Chancellor of the Exchequer's proposal respecting maintenance of criminal lunatics, 257.
Chancery lunatics, a letter on, by a suitor, 630.
Christianity and infidelity contrasted (in "the Closing Scene"), by the Rev. H. Neale, 161.
Circles, three, of life, Dr. Bergmann's theory of, 197.
Circulation, effects of, on the sensorial functions, 384.
Classes, labouring, progress of, 8.
Commissions in lunacy, Captain James Gordon, 152.
——— Miss Teresa Wakeman, 153.
Commissioners of lunacy, fifth report, 111.

Conduct of Col. Jack at Mallow, 415.
Confinement, solitary or separate, its effect on the mind, 259.
Confession of John Lynch, the murderer, 583.
Consciousness, the base of all mental operations, 398.
Crania, idiotic, and idiocy, 367.
Cretinism, *ib.*
Cranium, dimensions of, in idiots, cretins, &c., 372, 373, 378, 380.
Criminal lunatics, asylum for, 625.
Crichton, opinion of, concerning the post-mortem appearances of the insane, 349.
Cunning of young females ascribed by Dr. Laycock to ovarian excitement, 31, 33.
Dancing, prejudicial effects of, on young females, 38.
Delusions, how modified, 192.
——— nature of, explained by patients on recovery, 194.
Devay, Dr., on the premonitory symptoms of cerebral disease, 260.
Demoniacs, opinion of Bishop Œlisœus on, 202.
Diathesis, irritable, proclivity of, to mental disease, 122.
Diet, influence of, on the mind, 75.
Dietetics, mental, 89.
Diseases, cerebral, premonitory symptoms of, 260.
Diseases, nervous, Dr. Bennett on, 266.
Disposition changed by disease, 170.
Donelly, a lunatic, his evidence admitted on a trial for manslaughter, 284.
Dreaming (sleep and insanity), 461; Dr. Symonds on, 463, 477, 499; Dr. Fosgate on, 479, 482, 488, 495; Dr. Binns on, 485, 486, 489; Dr. H. Monro on the condition of the mind in, 124.
Dream, Coleridge's, 494.
Dreams, prophetic, 496.
Dyce Sombre, Mr., case of, 154.
Dysentery, insanity cured by an attack of, 381.
Earthquake, great, of Lisbon, supposed to have produced the catarrhal diathesis of the present time, 173.
Education, hygienic, importance of, to the children of excitable parents, 413.
Education of idiots, Dr. Poole on, 157.
Effects of opium and wine on the mind contrasted, 109.
Electro-biology, 355.
Emotion, mental, its influence in the production of diseases of the heart, 422.
England under Queen Victoria, contrasted with Rome under Augustus, 15.
Epidemics, mental aspects of, 171.
———, no memorials of, deserving credit earlier than the close of the fifteenth century, 173.
Epidemic dementia by imitation, 173, 174.
Epilepsy, observations on treatment of, 564, 565.
Escape of lunatics from asylums less numerous under diminished restraint, 144.
Exhibition, great, 1.
———, a fit study for the psychologist, 9.
———, the psychological interest of, pointed out by the Prince Consort, 10.
Evidence, medical, in cases of insanity, 574
——— in the case of M'Naughten, 576.
——— of a lunatic admissible in a court of justice, 436.
Faculties, special, highly developed in dreams, 499.
Febrile excitement may restore the idiotic to reason, 381.
Feelings have no memories, 403.
Feuchtersleben's opinion of the compound nature of insanity, 189.
Fœtus, progressive development of the brain in the, 399.
Fry, Mrs., early change of character in, 164.
Greding, Dr., remarks on his records of post-mortem appearances, 236.
Harmless lunatics, so called, remarks on, 551.
Heart, disease of, produced by mental emotion, 422.
Homicidal insanity, singular case of, 560.
House of Hapsburg, insanity of, 199.
———, genealogical table of, 200.
Howe, Dr., on the causes of idiocy, 369.
Hydrocephalus, case of, 459.
Hypnology (nervous sleep), Braid on, 356.
Idiots, education of, 157.
Idiocy, causes of, 368.
Idiots and cretins, distinction of, 367, 368.
Illegitimate children, proportional numbers of, in different states, 46.
Incomes, annual, of Chancery lunatics in 1848, 633.
Incubus of children, 481.
Insane, high rate of mortality among, 349, 457.
Insanity, moral, in young females, from ovarian excitement, treated by galvanism, 34.
——— physical, nature of, 119.
——— origin of, sometimes mental, sometimes physical, 189.
——— from solitary and separate confinement, 259.
——— ascribed by Dr. Monro to loss of nervous power, consequent on loss of vitality, 346.
——— comparative, of the sexes, 351.
——— statistics of, 85, 111, 112, 128, 129, 133, 134, 137, 140, 193, 200, 209, 210, 349, 350, 351, 352, 353, 430, 431, 433, 434, 435, 436, 525, 533, 534, 535,

537, 538, 539, 541, 542, 543, 544, 545, 546, 547, 548, 551, 553, 556, 559, 560, 561, 562, 563, 564, 566, 567, 569, 570, 572, 573.
——— homicidal, case of, 560
——— slight indications of, suggest vigilance, 407, 408.
——— moral, Dr. Browne on, 555.
Insane, mental manifestations and impulses of, 407.
——— kindness of the Irish towards, 415.
——— notions, polarity of, 485.
——— the, influence of religious consolation on, 552.
——— necessity of employment for, 557.
Instinct and reason, J. Q. Rumball on, 392
Instinct, definition of, 404.
——— and reason contrasted, 322, 404.
Intermarriage of relatives productive of idiocy, 369.
Ireland, private asylums of, 89.
——— public asylums, 83, 521.
——— asylum for criminal lunatics, 527.
——— number of pauper lunatics in, 85.
Johnson, W., M.B., Treatise of, on the Morbid Emotions of Women, noticed, 49, 50.
Judges, opinions of, on the admissibility of the evidence of lunatics, 447, &c.
——— remarks on the conduct of, towards medical witnesses, 575.
Jurisprudence, medical, of insanity, lectures on, by Dr. Jamieson, 187.
Lacing, tight, injurious effects of, 39.
Laycock, Dr. Thomas, Treatise on the Nervous Diseases of Women, 47.
Liability of a lunatic's estate for necessaries supplied, 297.
Life an immaterial principle and secondary agent, 397.
——— effects of insanity on duration of, 350.
Literature, German Psychological, 197.
Loudun's, Dr., hypothesis of the alteration of the atmosphere produced by the earthquake of Lisbon, 173.
Lunacy, state of, in England, 111.
Lunatic, evidence of a, in a trial for manslaughter, 283.
——— ruled by the judges to be admissible, 436.
Lunatics, number of, in England, 112.
——— "not dangerous to themselves or others," 551.
——— criminal, disposal of, 115.
——— central asylum for, 625.
——— pauper, in Ireland, 85.
——— Chancery, a letter on, by a suitor, 630.
Magpie, calculating powers of, 401.
Martineau, Miss, on the laws of man's nature and development, 303.
M'Naughten, case of, 576.
Meeting of the Association of Medical Officers of Hospitals for the Insane, 620.
Menstruation, first mean age of, in various climates, and different classes, 37.
Mental emotion, its influence in producing disease of the heart, 424.
Mesmeric manipulation, effects of, 356.
Metaphysics, modern, 317.
Mind, opinions of various philosophers, ancient and modern, on, 393.
——— and soul, not identical, 397.
Monro's, Dr., theory of insanity, 116, 341.
——— letter on public asylums for the insane of the middle classes, 594.
Moral insanity, Dr. Browne on, 555.
Mortality in lunatic asylums, 259, 457.
——— relative, of male and female lunatics, 259, 457.
Motives, false, of suicides, 453.
Murderer's confession, the, 583.
Nervous system, comparative anatomy of, 339.
Neurine, the universal concomitant of consciousness, 401.
Newfoundland dog, instinct of, 404.
Nola, sanitary precautions at, during the epidemic of 1815, 180.
Notes of a recent visit to some of the American lunatic asylums, 429.
Notices, miscellaneous, of various works, 300, 301, 460, 635.
Occupation or profession of suicides in France, 419.
Odours, sexual, 26.
Officers of Irish lunatic asylums, list of, 535.
Offspring, illegitimate, of the higher and middle classes, dangerous members of society, 46.
Opium, its effects on the mind, 108.
Organs, reproductive, the, influenced by the voice, 27.
Ovarian irritation, Dr. Laycock's treatment of, 34.
Oxford, Edward, present state of, 580.
Parents, imprudence of, in the management of children, 411.
Pestilences, epidemic, mental and moral effects of, 175.
Phosphorus deficient in the brains of idiots, 382.
Phthisis, frequency of, among the insane, 387.
Physiology, primary meaning of word, 469.
Pineal gland, Dr. Bergmann's theory of function of, 198.
Poems by a prisoner in Bethlem, 231.
Poole, Dr., on the education of idiots, 157.
Population, increase of, check on, among the higher and middle classes, 45.
Post-mortem examinations of the insane, 236, 385.
Powers, perceptive, of the inferior animals superior to man's, 401.

Propensity, morbid, to destroy offspring sometimes seen in human female, 33.
Property of lunatics, defective state of the law relating to, 115.
Prostitution, 43; Du Chatelet on, 44, 45.
Psychiatry, the causes of, 44; contributions towards a history of, 199.
Quincey's, De, confessions of an opium-eater, 108.
Reason and instinct, 322, 404.
Reason of the inferior animals, opinions on, of Burnett, Locke, Hancock, Good, and Addison, 320.
Reasoning, theory of, by S. Bailey, 503.
Reichenbach's "new imponderable," 357.
Report, the Fifth (for 1850), of the Commissioners of Lunacy, 111.
——— the Sixth General (for 1850), of District, Criminal, and Private Lunatic Asylums in Ireland, 521.
——— of the Board of Visitors of the Boston (U. S.) Lunatic Asylum *in re* Dr. Stedman, 152.
Reports of British Lunatic Asylums analyzed, 536 to 574.
Responsibility, moral, the nature of, 196.
Restraint, simple means of, 414.
Salaries of officers of Irish lunatic asylums, 535.
Scene, the closing, 161.
Scepticism, modern, 303.
Seduction a frequent cause of suicide in France, 254, 421.
Self-destruction sometimes hereditary, 195; when to be attributed to insanity, 195
Sensation, Dr. Wood on, 465.
Sensations in drowning, Dr. Adam Clarke's, 489.
Sensorium, influence of circulation on, 384.
Sentiments, last, of suicides, by Dr. A. Brierre de Boismont, 243, 448, 606.
——— general table of, arranged numerically, 244; good, 245; bad, 448; mixed, 606.
Sleep, dreaming, and insanity, 461; Dr. Fosgate on, 462, 500, &c.; Dr. Symonds on, 466, 475; of plants, 475; of hybernating animals, 476.
Sonnets, by Mr. Pearce, 235.
Spirits, philosophy of, in relation to matter, 210, 317.
Stays, female, remarks on, 39.
Statistics of mental derangement, in Germany, 209; in the Faroe Isles, 210; of Cretinism in Sardinia, 210; of suicide in France, 417.
Suicide, causes of, in France, 246, 421.
——— influence of age on, 418; influence of season on, 419; influence of geographical position on, 418.
——— mode of, influence by profession or occupations on, 420.
Suicides, profession or occupation of, 419.
——— religious feeling sometimes exhibited by, 253.
——— less frequent in asylums than formerly, 113, 144.
Sumbul in the treatment of epilepsy, 564.
Swift's, Dean, Hospital for Lunatics, 88.
Symptoms, premonitory, of cerebral disease, 260.
Syphiliphobia, Mr. Acton on, 456.
System, nervous, distinctive of the animal kingdom, 398.
Tables of cranial dimensions, 372, 373, 378, 380, 382.
Temperament, effect of, on the constitution of the mind, 374.
Theory of insanity, 116; of reasoning, 503
Trance-sleep, 476.
Trial of an attendant for the manslaughter of a patient in an asylum, 279.
Voice, sexual influence of, 28.
Wail, The Maniac's, 617.
Water, want of, in French asylums, 142.
Will, faculty of, always impaired in insanity, 190.
Will case, Dryden *v.* Fryer, 285.
Wilna, typhus in, during the retreat from Moscow, 176.
Window, Dr. Wood's, for asylums, 149.
Woman, psychological relations of, 18.
——— instinctive acuteness of the perceptive faculties in, 22.
Women, proportion of married to unmarried, in England, 42.

Savill & Edwards, Printers, 4, Chandos-street, Covent-garden.

